# Risky HIV sexual behavior and utilization of voluntary counseling and HIV testing and associated factors among undergraduate students in Addis Ababa, Ethiopia

**DOI:** 10.1186/s12889-017-4060-y

**Published:** 2017-01-25

**Authors:** Desalegn Woldeyohannes, Yehenew Asmamaw, Solomon Sisay, Werissaw Hailesselassie, Kidist Birmeta, Zinaye Tekeste

**Affiliations:** 1 Aklilu Lemma Institute of Pathobiology, Addis Ababa University, P.O. Box 1176, Addis Ababa, Ethiopia; 2 Department of Medicine, School of Medicine and Health Sciences, Addis Ababa Science and Technology University, P.O. Box 16417, Addis Ababa, Ethiopia; 3 Department of Medical Parasitology, Addis Ababa University, P.O. Box 1176, Addis Ababa, Ethiopia; 4 Department of Clinical, John Hopkins University-TSEHAI Project, P.O.Box 5606, Addis Ababa, Ethiopia; 5 Department of Public Health, Addis Ababa University, P.O. Box 1176, Addis Ababa, Ethiopia; 6 Department of Demography and Population study, Addis Ababa University, P.O. Box 1176, Addis Ababa, Ethiopia

**Keywords:** HIV, VCT use, Risky sexual behavior, Addis Ababa, Ethiopia, University students

## Abstract

**Background:**

HIV/AIDS is a major public health problem in Ethiopia. University students are often a young and sexually active group that is at risk of acquiring and transmitting HIV. We assessed risky HIV sexual behaviors and utilization of voluntary counseling and testing services among undergraduate students at Addis Ababa Science and Technology University, Ethiopia.

**Methods:**

A cross-sectional study was conducted between May and June, 2013. Standardized semi-structured self-administered questionnaire was used to collect data. Simple random sampling technique was use to select departments from each school. All students in the selected departments were the study participants. Data were entered into EPI-Info and analyzed using SPPS statistical packages. *P*-value < 0.05 was considered as statistically significant.

**Results:**

Of the total 602 students selected, an overall response rate of 557 (92.6%) were registered. Among the participants 361 (60%) were males. The student ages’ were ranged from 17 up to 25 years with mean age of 20.3 ± 1.6. Around 385 (64%) of them were in the age group of 17 up to 20 years. Among the study participants, 161 (26.8%) had sexual contact and the mean age of first sexual encounter was 17.4 (SD =2.3) years. About 443 (76%) of students knew that condoms can prevent Sexually Transmitted Infections (STIs). Among sexually active students, 74 (46%) had not used condom during first time sex. Among those responded, 488 (83.4%) had heard information about VCT; however, 52% had not ever used VCT service. The overall mean score of knowledge and attitude of students towards risk perception on HIV was around 66% and 57%, respectively. Students who enrolled in health science departments had almost three time more knowledge [AOR(95%CI) = 2.83 (1.67, 4.80)] and two and half times more favorable [AOR (95% CI) = 2.55 (1.60, 4.06)] attitudes towards HIV risk reduction strategies than students in non-health related departments.

**Conclusions:**

Some students were engaged in risky sexual behaviour even though they had heard about HIV/AIDS. The perception of risk for acquisition of HIV infection and utilization of VCT were low. HIV prevention and control strategies including education in the areas of HIV/AIDS as part of university programs curriculum, specially non-health students, and strengthening health institutions to provide youth-friendly VCT services for HIV with “know your HIV status” campaigns are strongly recommended.

## Background

Since 2000, around 38.1 million people have become infected with HIV and 25.3 million people have died of AIDS-related illnesses [1]. In addition to improved access to antiretroviral treatment and care in many regions of the world, AIDS epidemic claimed 1.2 million lives in 2014 alone and mainly occur in sub-Saharan Africa that account 66.6% of all people living with HIV [1]. Young people are particularly vulnerable to both acquiring and transmitting HIV and more than 50% of all new infections worldwide are among young people between the ages of 15 and 24 [2].

In sub-Sahara Africa, the majority of HIV transmission occurs through heterosexual intercourse, mother-to-child transmission and unsafe blood transfusion [1]. Additionally, in the absence of an effective vaccine and cure, voluntary counseling and testing has been used as an entry point, a gateway to various prevention and care interventions including antiretroviral treatment.

A study that was done in Uganda on sexual knowledge, attitudes and behavior among urban youth in 2003 revealed that about 43% of Ugandan young people have had sex by the age of 15 and nearly 70% of sexual activity among young people is unprotected [3]. Moreover, over 33% of boys and young men had slept with two or more partners in the previous three months and the majority had little or no knowledge on preventing HIV. Thirty five percent of those who knew that condom were protective used one the last time they had sex compared with 19% of those who hadn’t know . Over 33% of respondents did not know where to buy condoms. And 74% of youth knew that people who look healthy can still transmit HIV. In the same study, 51% of the participants thought they are at no risk when having unprotected sex with casual partners. Among 70% of youth who would like an HIV test, only 6% have had done the test [3].

When the HIV test was developed in mid 1980s, testing was intended to be accompanied by HIV counseling [4]. However, with the growing awareness of Human Immunodeficiency Virus/Acquired Immunodeficiency Syndrome (HIV/AIDS) and the recent availability of antiretroviral therapy (ART), the scope of and reasons for voluntary counseling and HIV testing (VCT) have broadened. VCT is a process by which an individual undergoes counseling to enable the youth to make an informed decision about being tested for HIV, assess their personal risk for HIV and develop a risk reduction strategy. The services are essential components of HIV prevention and care programs. However, initially many people were reluctant to be tested even if care and treatment were made available to them [4].

HIV/AIDS epidemic among youth is largely ignored and remains invisible to both young people themselves and to society as a whole. They are more likely to carry the virus for years without knowing that they are infected, consequently the epidemic spreads beyond high risk groups to the broader population of young people that making control harder and current data indicates that about 20% of young people aged 15–19 years (mainly secondary school students) are infected with HIV virus [5].

HIV voluntary counseling and testing (VCT) is now widely accepted as the cornerstone of HIV prevention programs in many countries because of its multiple benefits. Furthermore, VCT is the gateway to comprehensive HIV care and support including access to antiretroviral therapy [3]. Many people including the young do not seek VCT services until they develop symptoms of AIDS. Among the youth, barriers to VCT include lack of information, perception of low risk, lack of privacy and confidentiality, costs and laws that require parental consent [5].

In Ethiopia, according to the ministry of health (MoH), HIV prevalence was more pronounced in younger age groups of 15–30 years including a 8.6% of antenatal care (ANC) attendants in the same age groups were HIV positive. HIV counseling in Ethiopia began in the late 1980s with service expanding throughout 1990s, and it is reported that many people with HIV in Ethiopia do not know that they are infected [6].

A study conducted among high school student in Addis Ababa showed that 62% of the students who supported on utilization of condom at the time of sex answered that 42% of sexually active students reported using it on their first sexual encounter while only 28% said that they used it every time [7]. One study carried out to assess the perception of high school students on risks for acquiring HIV and use of VCT service reported that among sexually active students, 12 (40%) had sex with different persons within the last 6 months, 13 (43.3%) had ever used condom and 15 (50%) had used VCT service [8].

One study done among university students in Ethiopia reported practicing casual sex/ sex for any benefit with their first sexual partner and multiple sexual partners in the last 12 months were found to be the independent predictors of risks for STIs and/or HIV infection [9]. Therefore, the main objective of this study was to assess risky sexual behavior of university students towards HIV/AIDS and use of VCT service.

## Methods

### Study area and study period

The study was conducted from May to June 2013 in Addis Ababa City administration.

### Study design

Cross sectional study design.

### Study population

All regular students of Addis Ababa Science and Technology University were considered as the study population.

### Sample size determination

Sample size was determined based on assumptions including 95% confidence level, 4% margin of error (to increase degree of precision) and 15% for anticipated non-response rate of the respondents. Hence, a total of 602 participants were considered.

### Sampling procedure

Schools were identified based on their labeled departments in the University. At the same time, the study populations were also categorized from source population based on their schools. Then, Out of the total 23 departments, 15 departments were selected based on simple random sampling. The number of study subjects included in each department was proportional to their size. The students from the selected departments assembled in a room and were made to fill out a questionnaire in the presence of date collectors.

### Exclusion criteria

Students who were not attending their class at the time of data collection and those students who were learning in the evening were excluded from the study.

### Instrument of data collection and techniques

Data were collected using self-administered structure questionnaire. The questionnaire was prepared in English. Questions used to assess knowledge, attitude and practice of students towards HIV transmission and on VCT were adopted from different related studies. A Pre-test was conducted on 10% (60) of the students in the University. This helped us to verify the validity and reliability issues. The questionnaire was revised based on the findings of the pilot test.

### Data collection and quality control

Data collection was conducted by two graduate nurses. Data collectors received a half day training on issues concerning the questionnaire (on the objective of the study, the how of approaching the participants, how to administer and collect the questionnaires timely) was done. Consequently, the questionnaire was revised before data collectors were disseminated to collect data. Confidentiality of the study participants were kept during distribution and data collection periods. Above all, ethics, coding and entry were maintained throughout the process.

### Dependent variables

Knowledge and attitude of students towards HIV risk perception were considered as dependent variables.

### Independent variables

Age, sex, region, department, year of study and religion were considered as independent variables.

### Operational definitions

**Health Science Departments** include Public Health Officer and Nursing.

**Non-Health Science departments** include Basic Science and Engineering Departments.

Basic Science Departments: Industrial Chemistry, Computer Science and Information Technology, Ecobiology, Earth Science, Biotechnology).

Engineering Departments: Water Supply Engineering, Urban Planning and Design Engineering, Manufacturing Engineering, Food Processing Engineering, Environmental Engineering, Electro-Mechanical Engineering, Electrical and Electronics Engineering, Architecture Engineering.

### Risk

A situation in which an action will result in an outcome that is not known with certainty, but the set of possible outcomes and their associated probabilities are known or can be estimated.

### Behaviour

Various voluntary movements undertaken by the body in response to motives and decision related to HIV preventive methods.

### Perception risk

Students’ attitude towards perceiving themselves as susceptible to HIV infection.

### Data analysis

Questionnaires were checked for completeness. Partially completed questionnaires were excluded from analysis. The questionnaires were coded and the data entered in to EPI-Info version 2002 statistical package and analyzed by SPSS Version 16.0 package. Departments from which students are selected to participate were categorized into three broad themes (as Health Science, Basic Science and Engineering students). The score of one or zero was given based on the correct or wrong answer to individual knowledge and attitude questions. The knowledge score of 50.0% or above was graded as good knowledge and below this cut-off point as poor knowledge. The score of 50.0% and below was graded as unfavourable attitude and above this cut-off point as favourable attitude. Whereas students were categorized as with safe practice when they were involved in none of the risky practices. Collected data were summarized using frequency, percentages, and ratios. Chi-square (*χ*2), binary/ multiple logistic regression analyses were computed in order to assess the association and measure the level of significance of the association, respectively. Further, logistic regression was used to adjust for possible confounding factors. Results were reported using *P* < 0.05 level of statistical significance.

### Ethical considerations

Ethical approval was obtained from the Institutional Review Board of Addis Ababa Science and Technology University. Consent was requested and obtained from each student prior to the study. No personal identification was recorded on the questionnaire for ethical reason. The respondents had the right not to participate in or withdraw from the study at any stage.

## Results

### Socio-demographic characters

**Fig. 1:**
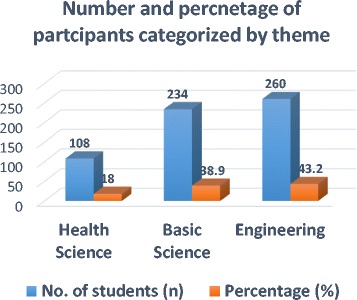
Number and percentage of participant students categorized by their thematic areas of field of study in Addis Ababa Science and Technology University, Addis Ababa, May-June 2013

A total of 602 students agreed to participate and completed the questionnaire. An overall response rate of 92.6% were reported. Whenever the number of students responded for a particular question is lees that 602 are shown in bracket next to each question. Participant students were in the Health Science 108(18%), Basic Science 234(38.9%) and Engineering 260 (43.2%) fields of studies (Fig. 1).

The study revealed that out of the total participants 361 (60%) of the participants were males, and the mean age of the participants was 20.3 ± 1.6 which ranges from 17 to 25 years old. The majority 385 (64%) of the participants were in the age group of 17 and 20 years. Most of the students 263 (43.7%) were from Amhara Ethnic group followed by Oromia 170 (28.2%). Orthodox 408 (67.8%) and Protestant 94 (13.8%) religion followers were the dominant religions among the participants (Table 1).

**Table 1:** Socio-demographic characteristics of Addis Ababa Sciences and Technology University Students, Addis Ababa, May to June 2013

Characteristics	Frequency	Percentage
Age group (Years)		
17-20	385	64.0
21-25	209	34.7
26^+^	8	1.3
Sex		
Male	361	60
Female	241	40
Ethnicity		
Amhara	263	43.7
Oromo	170	28.2
Tigray	125	20.8
Other	44	7.3
Class year		
First	175	29.1
Second	427	70.9
Religion		
Orthodox	408	67.8
Protestant	94	15.6
Muslim	82	13.6
Catholic	9	1.5
Other	9	1.5

### Students knowledge on HIV infection

Two hundred forty three (42%) of the participants believed that HIV can be transmitted through kissing and sharing different equipment. Among 576 study subjects who responded, 66 (11.5%) of individuals did not know about Sexual Transmitted Diseases (STDs), and further 51 (10%) of study subjects also didn’t have the knowledge of HIV as one of STDs. The overall mean score of knowledge of students towards HIV was 65.9% (Table 2).

**Table 2:** Knowledge on HIV/AIDS in Addis Ababa Sciences and Technology University Students, Addis Ababa, May to June 2013

Knowledge	Frequency	Percentage
Do you think Condoms can prevent STDs? (*N* = 581)		
Yes	443	76.2
No	134	23.1
Probably	4	0.7
Can HIV transmit through kissing and sharing equipment(*N* = 583)		
Yes	243	41.7
No	307	52.6
I don’t know	33	5.7
Is HIV STDs? (*N* = 491)		
Yes	440	89.6
No	51	10.4
Can HIV transmit through breast feeding (*N* = 563)		
Yes	311	55.2
No	252	44.8
Know about STDs (*N* = 576)		
Yes	510	88.45
No	66	11.5

### Students’ risk perception towards HIV infection

Participants’ perception on their risk of acquiring HIV infection was asked. Among the 560 students who replied, the result indicated that 199 (35.5%) of them believed to have risk and 361 (64.5%) not. The proportions of students who perceived themselves at risk of contracting HIV were not similar for both sexes (123 (61.8%) for male and 76 (38.2%) for female) and moreover, students enrolled in the non-health departments reported to have high risk 171 (85.9%) for acquiring HIV when compared to health students 28 (14.1%).

### Students’ attitude towards HIV infection

Among the 582 participants who responded, 83 (14.19%) did not believe that HIV is severe, and it affects more youth than other group of population. And, 116 (20.7%) of students did not believe that maintaining virginity before marriage doesn’t help for the prevention of HIV/AIDS. The overall mean score of attitude of students towards risk perception on HIV was 56.6% (Table 3).

**Table 3:** Attitude on HIV/AIDS at Addis Ababa Sciences and Technology University Students, Addis Ababa, May to June 2013

Character	Frequency	Percentage
Do you think HIV/AIDS is dangerous and has no cure? *N* = 579		
Yes	458	79.1
No	94	16.2
I do not know	27	4.7
Do you think HIV/AIDS and other STDs cannot transmit while having sex with known person? *N* = 571		
Yes	152	26.6
No	372	65.2
I don’t know	47	8.2
Do you believe commercial sex workers responsible for HIV transmission? *N* = 565		
Yes	355	62.8
No	210	37.2
Do you think you are at risk of acquiring HIV Infection? *N* = 560		
Yes	199	35.5
No	361	64.5
What is your chance of acquiring HIV infection? *N* = 571		
No	151	26.4
Low	308	53.9
Medium	22	3.9
High	45	7.9
I do not know	45	7.9
Do you believe only those people who lead immoral lives will get HIV? *N* = 567		
Yes	188	33.2
No	379	66.8
AIDS patients should be isolated for the safety of others: *N* = 586		
Agree	185	31.6
Disagree	401	68.4
Do you think HIV/AIDS is sever and more affects youth? *N* = 582		
Agree	499	85.7
Disagree	83	14.3
Women are more responsible than men for prostitution: *N* = 566		
Agree	394	69.6
Disagree	172	30.4
What is your perception about maintaining virginity for prevention of HIV/AIDS? *N* = 560		
It prevents HIV	300	53.6
It doesn’t prevent HIV	116	20.7
No response	144	25.7

### Sexual behaviour of students

One hundred sixty one (26.8%) of the respondents had sexual experience. Out of whom, 33 (20.5%) were females and the rest 128 (79.5%) were males. The students’ age to start sex was ranged from15 up to 19. With regards to use of condom, 74 (46%) of respondents claimed that they did not use at the first time of intercourse (Table 4). Among those students who responded to question about use of condom during sexual intercourse, 25 (21.4%) did not use condom during sex (Table 4). Among those who responded to the question for whether they had more than one sexual partner 40 (28.2%) of them said yes. The main reasons mentioned by students for having multiple sexual partners include to satisfy sexual desire 33 (82.5%), due to cultural reasons 6 (15%), due to seeking to have more children 1 (2.5%) and economic reason 1 (2.5%). Among male students who started sex, 23 (18%) of them ever had sex with commercial sex workers (Table 4).

**Table 4:** Students sexual behavior towards HIV infection at Addis Ababa Sciences and Technology University Students, Addis Ababa, May to June, 2013

Character	Frequency	Percentage
Have you ever had sex? *N* = 602		
Yes	161	26.8
No	441	73.2
What was your age when having first sex? *N* = 161		
< 15	16	9.9
15-19	99	61.5
20-24	41	2.5
> 24	5	3.1
Did you use condom during first time sex? *N* = 161		
Yes	87	54
No	74	46
What was the reason for first sex? *N* = 161		
Voluntary	155	96.3
Rape	6	3.7
Who was your first sex partner? *N* = 161		
Forcefully done	3	1.9
Fiancé	10	6.2
Boy/girlfriend	141	87.6
Commercial sex worker	7	4.3
Did you practice sex under the influence of: *N* = 161		
Alcohol	38	23.5
Drugs	3	1.9
Money/other benefit	46	28.6
No alcohol/drug/benefit	74	46.0
How many sex partners do you have currently? *N* = 142		
1	102	71.8
> 1	40	28.2
Reason for having more than one partner: *N* = 40		
To satisfy sexual desire	33	82.5
For cultural reasons	6	12.5
To get more children	1	2.5
Economic reasons	1	2.5
What is the age difference with your sex partner? *N* = 134		
< 5 years	95	70.9
≥ 5 years	39	29.1
Have you ever had sex with Commercial Sex Workers? (FOR MALES ONLY) *N* = 128		
Yes	23	18
No	95	82
Do you use condoms when having sex? *N* = 117		
Yes	92	78.6
No	25	21.4
When do you use condom? *N* = 103		
At first sex only	17	16.5
Always	86	83.5
Do you use Alcohols/drug? *N* = 432		
No	332	76.9
Rarely	77	17.8
Commonly	23	5.3
Do you see sex films? *N* = 517		
Yes	243	47.0
No	274	53.0

Students enrolled in health departments were three times more knowledgeable (*P* < 0.05) [AOR (95% CI) = 2.83 (1.67, 4.80)] in risks involved in HIV transmission than students in non-health departments (Table 5). Moreover, students enrolled in health departments had almost two and half times more favourable attitude [AOR (95% CI) = 2.55 (1.60, 4.06)] (*P* < 0.05) towards HIV prevention methods than students in non-health departments (Table 6). However, variables like sex, age, study year, religion and ethnicity did not show any association with both knowledge and attitude of students (*P* > 0.05) towards HIV prevention strategies.

**Table 5:** Association of socio-demographic characteristics and knowledge of AASTU Students towards HIV infection May to June 2013

Variables	Good knowledge		OR (95%CI)	OR (95%CI)	*P*-value
	Yes (%)	No (%)	Crude	Adjusted	
Age					
17-20	262(43.5)	123(20.4)	1.00		
21-25	128(21.3)	81(13.5)	0.74 (0.52, 1.05)		
+ 25	7(1.2)		3.3 (0.4, 27.0)		
Sex					
Male	247(41.0)	91(15.1)	1.00		
Female	150(24.9)	114(18.9)	1.3 (0.93, 1.85)		
Ethnicity					
Oromia	108(17.9)	62(10.3)	1.00		
Amhara	177(29.4)	86(14.3)	1.18 (0.79, 1.77)		
Tigray	87(14.5)	38(6.3)	1.31 (0.80, 2.15)		
Other	25(4.2)	19(3.2)	0.75 (0.38, 1.49)		
Department					
Non-health	308(51.2)	186(30.9)	1.00	1.00	0.001*
Health	89(14.8)	19(3.1)	2.83 (1.67, 4.80)*	2.83 (1.67, 4.80)*	
Year of study					
Year I	115(19.1)	60(10)	1.00		
Year II	282(46.8)	145(24.1)	1.0(0.70, 1.47)		
Religion					
Orthodox	273(45.3)	13.5(22.4)	1.00		
Muslim	49(8.1)	33(5.5)	0.73 (0.47, 1.39)		
Catholic	5(0.8)	4(0.7)	0.62 (0.16, 2.34)		
Protestant	65(10.8)	29(4.8)	1.11 (0.68, 1.80)		
Others	5(0.8)	4(0.7)	0.62 (0.16, 2.34)		

* Significant *P* < 0.05 level

**Table 6:** Association of socio-demographic characteristics and attitude of AASTU Students towards HIV infection May to June 2013

Variables	Favorable attitude		OR (95%CI)	OR (95%CI)	*P*-value
	Yes (%)	No (%)	Crude	Adjusted*	
Age					
17-20	223(37.0)	162(26.9)	1.00		
21-25	112(18.6)	97(16.1)	0.84 (0.60, 1.18)		
+25	6(1.0)	2(0.3)	2.18 (0.43, 11.0)		
Sex					
Male	212(35.2)	149(24.8)	1.00		
Female	129(21.4)	112(18.6)	1.23 (0.89, 1.72)		
Region					
Oromia	85(14.1)	85(14.1)	1.00		
Amhara	161(26.7)	102(16.9)	1.58 (0.70, 1.77)		
Tigray	72(12.0)	53(8.8)	1.36 (0.85, 2.16)		
Other	23(3.8)	21(3.5)	1.10 (0.56, 2.13)		
Department					
Non-health	261(43.4)	233(38.7)	1.00	1.00	0.002*
Health	80(13.3)	28(4.7)	2.55 (1.60, 4.06)*	2.55 (1.60, 4.06)*	
Year of study					
Year I	99(16.4)	76(12.6)	1.00		
Year II	242(40.2)	185(30.7)	1.0(0.70, 1.43)		
Religion					
Orthodox	231(38.4)	177(29.4)	1.00		
Muslim	44(7.3)	38(6.3)	0.89 (0.55, 1.43)		
Catholic	4(0.7)	5(0.8)	0.61 (0.16, 2.32)		
Protestant	56(9.3)	38(6.3)	1.13 (0.72, 1.78)		
Others	6(1.0)	3(0.5)	1.53 (0.38, 6.21)		

* Significant *P* < 0.05 level

### Knowledge, attitude and practice towards VCT

Among those students who responded to the question, 488 (83.4%) heard about VCT services. Majority of the participants 510 (89.9%) thought that getting VCT service is necessary. But, 210 (36%) of participants did not know about the existence of the service at the University (Table 7).

**Table 7:** Knowledge, attitude and practice on VCT at Addis Ababa Sciences and Technology University Students, Addis Ababa, May to June, 2013

Knowledge		
Character	Frequency	Percentage
Do you heard information about VCT (*N* = 585)		
Yes	488	83.4
No	97	16.6
Source of information (*N* = 585)		
Mass media	250	42.7
Newsletter	150	25.6
Colleague	100	17.1
Health professionals	70	12.0
Others	15	2.6
Knowing the presence of VCT provision center around (*N* = 582)		
Yes	372	64
No	210	36
Perceived importance of getting VCT (*N* = 577)		
Important	542	93.9
Not-important	35	6.1
Attitude		
Do you agree that getting HIV blood test would provide safety for others? *N* = 581		
Agree	479	82.4
Disagree	52	8.9
Neutral	50	8.7
Do you think VCT is necessary? (*N* = 567)		
Yes	510	89.9
No	57	10.1
Did VCT test help you in any way (e.g. alleviating your anxiety?) *N* = 264		
Agree	202	76.5
Disagree	24	9.1
Neutral	38	14.4
Preferable ways of getting HIV test result (*N* = 578)		
Face to face	468	81.0
Secretive letter	54	9.3
Telephone	29	5.0
From relative or partner	18	3.1
Others	9	1.6
When do you think one should get VCT service (*N* = 561)		
At any time	459	81.9
While feeling sick	58	10.3
Only when involved with many sexual partners	23	6.0
When ready for marriage	13	2.4
Others	8	1.4
Practice		
Have ever used VCT (*N* = 573)		
Yes	275	48
No	298	52
Are you willing to undergo VCT(*N* = 589)		
Yes	457	77.6
No	132	22.4
Purpose of getting VCT (*N* = 288)		
For marriage	83	31.7
To know their Sero-status	75	28.6
To prevent transmission of the virus to the fetus	65	24.8
To confirm to friends	39	14.9
Reason for not using VCT(*N* = 216)		
Never had sexual intercourse before	114	52.8
Fear of stigma by the society	50	23.1
Fear of stress due to the virus	29	13.4
Other	23	10.7

Out of 589 students who responded to the question, 457 (77.6%) were willing to be tested for HIV if they are asked to while the rest 132 (22.4) were not (Table 7). Moreover 468 (81%) of students wanted to hear their test result on face to face basis. On the other hand, among reasons mentioned 114 (52.8) and 50 (23%) of participants were not ready to take the VCT service because of the absences of previous sexual intercourse and fear of stigma by the society, respectively. A total of 479 (82.4%) respondents agreed that getting HIV blood test would provide safety for others (Table 7).

With regards to timing for testing, majority of the students 459 (81.9%) said that one should undergo VCT at any time, 58 (10.3%) thought when an individual falls ill, 23 (6.0%) thought when an individual having many sexual partners and 13 (2.4%) agreed just before marriage. Moreover, among the respondents who had used VCT 202 (76.5) believed that HIV test helps in alleviating anxiety and the rest 24(9.1%) disagreed (Table 7).

Out of 573 students, only 275 (48%) ever used VCT. Reasons mentioned to undergo VCT were, 83 (31.7%) did it for marriage, 75 (28.6%) to know ones sero-status, 65 (24.8%) to prevent transmission of virus to the fetus, and 39 (14.9%) just to confirm to friends Among reasons mentioned not to use VCT, no experience of sexual intercourse was the highest 114 (52.8%) and 50 (23%) of them mentioned feared stigma by the society if found positive (Table 7).

## Discussion

Overall, the result from our study demonstrated that the students had a moderate level of HIV/AIDS knowledge, with an average score of 66%. This result is similar to studies conducted among students in Ghana and Yemen [10, 11].

Our study revealed that around 42% participants thought that HIV could be transmitted through kissing and sharing of different equipment. Similar studies carried out in Lao People’s Democratic Republic and Yemen [11, 12] which supported our findings.

Another interesting finding of the current study was that almost 90% of the study subjects knew HIV as one of the STDs and over 55% participants were tested for HIV. This finding is consistent with other studies conducted in Hawasa Town and Gambella Region of Ethiopia [13, 14].

In this study, students enrolled in health departments were almost three times more knowledgeable [AOR (95% CI) = 2.83 (1.67, 4.80)] than students in non-health departments. This could be better explained due to the fact that curriculum of health science programs incorporated facts about infectious diseases in general and HIV/AIDS in particular. Similar findings were reported in a questionnaire based study to assess HIV/AIDS knowledge, attitude and behaviors of Chinese students in China which supported the finding that students majoring in medicine had more knowledge than non-medical students [10].

Overall, respondents’ attitude towards HIV/AIDS infection was 75.5%. Moreover, attitude of respondents about people who live with HIV were moderately positive with the average mean score of 66.4%. Studies conducted in Gambella in Ethiopia, Nigeria and Kerala University in India [14–16] showed similar results.

Students enrolled in health departments had almost two and half times more favorable attitude [AOR (95% CI) = 2.55 (1.60, 4.06)] towards HIV prevention methods than students in non-health departments. However, in comparative study [17] conducted among undergraduate students at Addis Ababa University of Ethiopia showed that there was no association between students’ attitude towards HIV prevention methods and their departments’ type (health science vs non-health science). In our study, the absence of association between attitude of students by ethnicity and by religion might be linked with sharing of the same cultural values by the students due to their social integration in the campus, which influences them to have similar attitude towards HIV prevention strategies.

Our study revealed that 78.6% of the participants used condom whenever they practice sexual intercourse, and around 18% of participants practiced sex with commercial sex workers. Similar report was obtained in researches conducted at Nigeria, Yemen and China [10, 11, 16].

The unwillingness of students to take HIV test in the study could be attributed to fear, anxiety stigma and discrimination which are linked to HIV/AIDS. Fear of stigma had shown to influence young adults to become less likely to engage in preventive behaviors like taking VCT service [10, 16].

The current study revealed that from the total 26.8% of the students had a history of sexual intercourse. Of these, 54%) of the students said they were using condoms during their first sex. Almost 8414.3% of students were using condoms regularly when they had sex with casual partners. This finding disagree with the result reported in Lao People’s Democratic Republic [12].

In our study, 83.4% of the students had heard about VCT service. The most common benefits of VCT mentioned were for marriage (31.7%), and to know HIV sero-status (28.6%). About 53.5% of the respondents had favorable attitude towards VCT service. The majority (77.6%) of the students were willing to take HIV test whether they did it or not in the past. Among those who had no desire for VCT, 52.8% mentioned it due to the absence of previous sexual intercourse followed by the fear of stigma by the society (23.1%). Similar results had been reported by studies done in Ethiopia and in Kenya [18–20].

## Conclusions

Almost two-third of the students were knowledgeable on HIV and only 56.6% of the respondent had favorable attitudes towards its prevention methods. Students were involved at different stages of risk for acquiring HIV infection. Students of health department had significant knowledge on HIV and attitude toward its prevention as compared from other department of students. In general, majority of the students had not used VCT services but were willing to use VCT service if contacted. It is recommended to design HIV prevention and control strategy including scaling up of VCT services at university level for increasing students’ awareness about HIV especially for those students who are in non-health related departments. And, future similar studies using qualitative methods are also highly recommended.

## Abbreviations

AASTU: Addis Ababa Science and Technology University
AIDS: Acquired Immunodeficiency Syndrome
ART: Anti Retroviral Therapy
CI: Confidence Interval
HIV: Human Immunodeficiency Virus
OR: Odds Ratio
SPSS: Statistical Package for Social Sciences
STDs: Sexually Transmitted Diseases
STIs: Sexually Transmitted Infections
VCT: Voluntary Counseling and Testing
WHO: World Health Organization
